# Protein Kinase A Regulates the Cell Cycle to Affect the Induction Rate in the Parthenogenetic Reproduction of the Silkworm, *Bombyx mori*

**DOI:** 10.3390/cells14110793

**Published:** 2025-05-28

**Authors:** Fang Xu, Wei Yu, Chenkai Ma, Chengjie Hu, Chunguang Cui, Xin Du, Jine Chen, Linbao Zhu, Shaofang Yu, Xingjian He, Yongqiang Wang, Xia Xu

**Affiliations:** 1 College of Life Sciences and Medicine, Zhejiang Sci-Tech University, Hangzhou 310018, China; 2 Institute of Sericulture and Tea, Zhejiang Academy of Agricultural Sciences, Hangzhou 310021, China

**Keywords:** PKA, enzyme activity, cell cycle, parthenogenesis, *Bombyx mori*

## Abstract

Protein kinase A (PKA), commonly referred to as cAMP-dependent protein kinase, exists as a heterotetramer composed of two catalytic (C) and regulatory subunits (R). This versatile kinase exhibits regulatory functions in various biological processes including growth, division, and differentiation. Although PKA is well established as a master regulator of oocyte maturation across species, its functional role in insect parthenogenesis has remained enigmatic. Here, we systematically investigated the regulatory effect of PKA in the induction of parthenogenesis in model lepidopteran *Bombyx mori*. Our findings demonstrated an inverse correlation between PKA activity and parthenogenetic induction efficiency in silkworms. Notably, PKA activation resulted in delayed embryonic development, whereas *PKA-C1* knockdown disrupted normal cell cycle progression. These results indicated that maintaining appropriate PKA activity is essential for ensuring proper cell division process, especially in the successful induction of silkworm parthenogenesis. The evolutionary conservation of PKA across species, coupled with its critical regulatory role in parthenogenesis, positions this kinase as a promising molecular target for breeding design. Our findings establish a foundation for developing silkworm strains with enhanced parthenogenetic capacity through PKA modulation, thereby facilitating the preservation of elite production traits. These results provide novel mechanistic insights into parthenogenesis while demonstrating the potential application of PKA regulation in both genetic studies and breeding programs.

## 1. Introduction

Protein kinase A (PKA) is an evolutionarily conserved serine/threonine kinase that functions as a primary effector of cyclic adenosine monophosphate (cAMP) signaling [1]. PKA is heterotetramer and consists of two catalytic and regulatory subunits [2,3]. The R subunits of PKA contain cAMP-binding domains: the C subunit that releases enzyme activity when intracellular cAMP levels are elevated; conversely, when regulatory and catalytic subunits remain bound, PKA remains inactive [4]. PKA is ubiquitously expressed across various tissues and exerts pleiotropic regulatory effects upon activation [5,6]. As a central signaling mediator, activated PKA phosphorylates diverse substrate proteins [7], thereby modulating a broad spectrum of cellular processes including but not limited to gene transcription [8], energy metabolism [9], cell proliferation and differentiation [4], cell cycle progression [10,11], and programmed cell death in virtually all tissue types [12].

PKA not only promotes sperm capacitation by modulating ion channels and enzyme activity [13] but also regulates oocyte maturation and meiotic progression through the cAMP signaling pathway [13,14]. Notably, changes in PKA activity directly influence the activation state of maturation-promoting factor (MPF). This regulation occurs through the precise phosphorylation of cell cycle regulators (Wee1, Cdc25) that dictate MPF activation states, ultimately determining meiotic arrest (MII) or completion [15,16]. In insects, PKA similarly exhibits crucial regulatory functions: in the cotton bollworm (*Helicoverpa armigera*), PKA modulates ecdysteroid biosynthesis via the cAMP/PKA/CREB signaling pathway, thereby influencing developmental and metamorphic processes [17]. In *Drosophila*, PKA mutations disrupt microtubule polarity in oocytes, impairing normal oocyte development [18]. The amphibian *Xenopus system* further demonstrates PKA’s evolutionary versatility, where regulatory subunit injection paradoxically promotes maturation through MPF activation [19]. These findings collectively highlight PKA as a highly evolutionarily conserved protein kinase that, through intricate signaling networks (such as the cAMP/PKA/CREB pathway and MPF activation mechanisms), serves as a central regulator in key physiological processes—including germ cell maturation, hormone synthesis, and developmental metamorphosis—across diverse species.

Parthenogenesis represents an exceptional reproductive mode wherein unfertilized oocytes undergo complete embryonic development to form viable offspring [20]. In lepidopteran model *Bombyx mori*, this phenomenon occurs naturally at minimal frequencies (~0.003%) [21,22]. Artificial induction methods, particularly thermal stimulation (46 °C for 18 min), can significantly enhance parthenogenetic development and yield exclusively female progeny [23,24]. Cytological hypothesis demonstrated that this treatment overcomes meiotic arrest at metaphase I, triggering an equational division that mimics mitotic processes and yields genetically identical clonal offspring [25,26].

While we have previously established that the activity dynamics of maturation-promoting factor (MPF) and its downstream effector cyclin-dependent kinase 2 (Cdk2) critically regulate parthenogenetic induction rates in silkworm [27,28], the upstream regulatory role of PKA in this process remains poorly understood. Notably, although thermally induced parthenogenetic strains have been successfully implemented in sericultural production [29], the molecular mechanisms governing this reproductive mode—particularly the hierarchical relationship between PKA signaling and the MPF-Cdk2 cascade—require systematic elucidation. This knowledge gap presents a key frontier in reproductive biology, as resolving PKA’s regulatory logic could (1) optimize existing induction protocols, (2) enable genetic enhancement of parthenogenetic efficiency, and (3) provide evolutionary insights into asexual reproduction across taxa.

Here, we elucidated the crucial regulatory role of PKA on parthenogenesis induction in silkworm. The results demonstrated a significant negative correlation between PKA activity and parthenogenetic induction efficiency. Our functional analyses demonstrated that PKA activity modulation exerts bidirectional control over parthenogenetic development in silkworm. PKA activity upregulation caused embryonic developmental delays, while PKA deficiency led to cell cycle dysregulation. It established that maintaining PKA activity homeostasis was essential for normal cell division and critical for successful parthenogenesis induction. Evolutionary analysis highlighted the high conservation of PKA across species, underscoring its universal role as a key regulatory node in reproduction. These discoveries not only clarified the molecular mechanisms underlying PKA-mediated parthenogenesis in silkworms. PKA’s evolutionarily conserved nature provided novel insights for reproductive regulation studies in other species. These also demonstrated considerable potential for applications in genetic improvement and breeding technology innovation.

## 2. Materials and Methods

### 2.1. Silkworm and Cells

The parthenogenetic lines (PLs, Wu9, and Wu14) were provided by the Zhejiang Academy of Agricultural Sciences. The PLs have been stably maintained for over 30 generations through warm bath induction (46 °C, 18 min) [23]. Rearing conditions were as described previously [27,28]. The BmN cell line, originally derived from *B. mori* ovarian tissue [30]. BmN cells provide unique advantages for insect biotechnology due to their immortalized cell system, genetic stability, and compatibility with modern molecular tools including CRISPR/Cas9-based genome engineering. Cells were cultured on 25 cm^2^ Petri dishes at 27 °C in TC-100 medium (LVN1013, Livning, Beijing, China) containing 10% fetal bovine serum (FBS).

### 2.2. Artificially Induced Parthenogenesis

Twelve hours after eclosion, unfertilized eggs were subjected to parthenogenetic induction [23]. The unfertilized eggs were immersed in a warm bath at 46 °C for 18 min. Subsequently, these were transferred to a water bath at room temperature of 25 ° C for rapid cooling for 3 min. Successful parthenogenetic activation was as described previously [27,28]. Developmental outcomes were quantified using two parameters: pigmentation rate (%) = (pigmented/total eggs) × 100, hatching rate (%) = (hatched/total eggs) × 100. Each experimental group included 45 moths for statistical analysis. Pigmentation indicated the initiation of embryonic development, and hatching indicated the completion of the whole process of embryonic development into an individual.

### 2.3. Protein Structure and Phylogenetics Analysis

The online software Uniprot for sequence annotation (https://www.uniprot.org/, accessed on 20 January 2025) and Swiss-model for 3D modeling (https://swissmodel.expasy.org/, accessed on 20 January 2025) [31]. E-value cutoff for BLASTP2.14.0 was <1 × 10^−10^. Phylogenetic analysis was then performed using the neighbor-joining method in MEGA 11 [32], employing the Poisson correction model for evolutionary distance calculation and assessing node support through 1000 bootstrap replicates, followed by visualization refinement using the Chiplot0.1.0 (https://www.chiplot.online/, accessed on 20 January 2025) [33].

### 2.4. RNA Isolation, cDNA Synthesis, and qPCR Analysis

Trizol^®^ reagent (Invitrogen, Carlsbad, CA, USA) was used to isolate total RNAs. Complementary DNA (cDNA) synthesis conditions were as described previously [27,28]. Quantitative real-time PCR (qRT-PCR) analyses were as described previously [27,28,34]. The specific primers used were listed in Table 1.

### 2.5. Enzyme Activity Regulation and Detection

PKA activity modulation was performed using synchronized healthy silkworm pupae (consistent pupation time) maintained at 25 °C and 80% RH incubator. PKA activity were modulated by activator (HY-B0764, MCE, Atlantic City, NJ, USA) and inhibitor (HY-15979A, MCE). The activator Bucladesine sodium salt (Dibutyryl-cAMP sodium salt, C_18_H_23_N_5_NaO_8_P) was a stable cAMP analog that activates PKA by elevating intracellular cAMP levels. Its mechanism involved mimicking endogenous cAMP to bind PKA regulatory subunits, thereby releasing catalytic subunits and enhancing PKA activity. The inhibitor H-89 dihydrochloride (C_20_H_22_BrCl_2_N_3_O_2_S) specifically targeted PKA by competitively binding to the ATP-binding catalytic subunits, preventing ATP binding and subsequent substrate phosphorylation. These compounds have been validated in insects and other animals, showing dose-dependent PKA activity modulation [35,36,37,38,39,40]. Different concentrations were configured as described previously [27,28], based upon which three appropriate concentrations (0.1 µM, 1 µM, and 5 µM) were finally set determined based on preliminary experiment. The administered injection was consistently 6 µL. Injection methods and enzyme activity assays were performed as previously described [27,28].

### 2.6. Observation of Ovariole and Embryo

The female moth had eight ovarioles. We observed the morphology by dissection 12 h after emergence. At the same time, we dissected and observed the continuous developmental morphology of the embryos. Dissection and photography were performed as described previously [27,28].

### 2.7. mRNA Synthesis and Cell Transfection

‘GGACAACTCTAACTTGTACATGG’ and ‘GGTTGATCAGCGAAAAAGGGCGG’ were designed single-guide RNA (sgRNA) target sites. The sgRNA templates were synthesized using T7 as described previously [41]. mRNAs synthesis and cell transfection were as described previously [28].

### 2.8. Cell Viability and Cycle Assay

Forty-eight hours after transfection, 100 µL cell suspension per well were added to a 96-well plate containing 10 µL of CCK-8 solution (40203ES60, YEASEN, Shanghai, China) in triplicate. Incubated at 27.5 °C for 2 h and measured absorbance at 450 nm using microplate reader to reflect the cell viability and proliferation. The cell cycle analysis was used, and the cell cycle and apoptosis analysis kit (C1052, Beyotime, Shanghai, China) were as described previously [28].

### 2.9. Statistical Analysis

Student’s two-tailed *t*-test analysis in GraphPad Prism 8.3.0. Each treatment included three independent replicates. Data were presented as means ± SEM (standard error of the mean).

## 3. Results

### 3.1. Protein Structures of PKA in Silkworm

**Figure 1:**
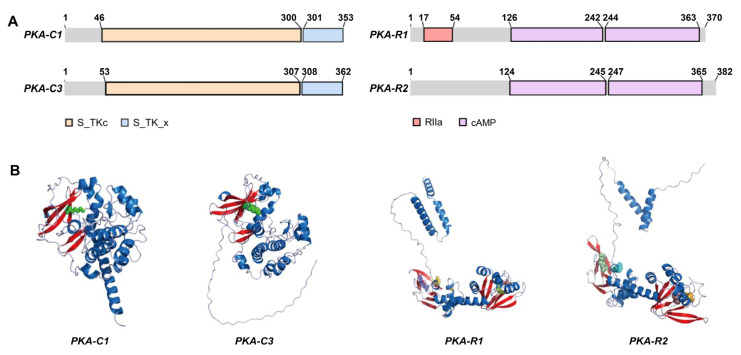
Predicted protein. (**A**) Functional domain. (**B**) Tertiary structure. PKA-C1 and PKA-C3 were catalytic subunits; PKA-R1 and PKA-R2 were regulatory subunits. S_TKc and S_TK_x regions were the serine/threonine protein kinases catalytic domain; RIIα region was the regulatory domain; cAMP region was the cAMP binding domain. Blue was α-helices; red was β-folded; gray was random coils.

In silkworm, we successfully identified and functionally characterized the catalytic and regulatory subunits of protein kinase A (PKA) (Figure 1). The analysis of putative structural revealed that both catalytic subunits PKA-C1 and PKA-C3 contain conserved S_TKc and S_TK_x domains, which facilitate the phosphorylation in specific substrate peptides. The regulatory subunits PKA-R1 and PKA-R2 each possess two cAMP binding sites. These binding sites play crucial roles in both PKA dissociation and activation, with cAMP binding exhibiting strong positive cooperativity in the enzyme activation process.

### 3.2. Evolutionary Conservation of PKA Proteins

**Figure 2:**
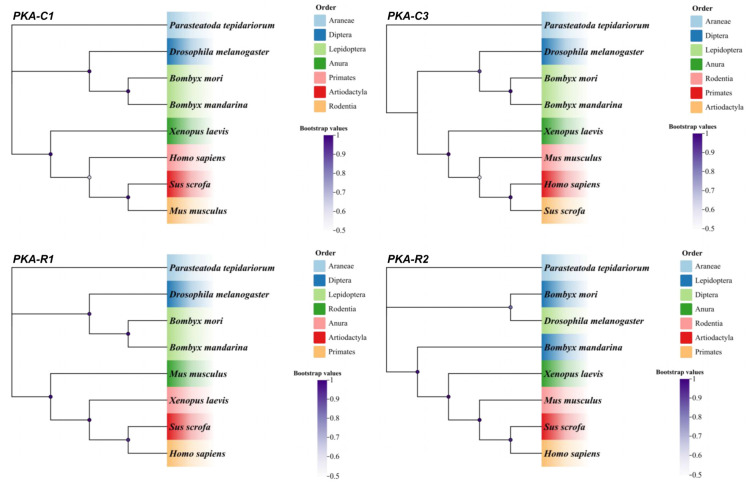
The phylogenetic relationship analysis. PKA-C1 and PKA-C3 were catalytic subunits; PKA-R1 and PKA-R2 were regulatory subunits.

The representative sequences evaluated were from Lepidoptera *(Bombyx mori*, *Bombyx mandarina*), Diptera (*Drosophila melanogaste*), Anura (*Xenopus laevis*), Primates (*Homo sapiens*), Artiodactyla (*Sus scrofa*), Rodentia (*Mus musculus*), and Araneae (*Parasteatoda tepidariorum*). The results indicated that these proteins were highly conserved (Figure 2). These data indicate that the functional insights from silkworm studies may extend to diverse taxonomic groups. The sequence numbers are listed in Table 2.

### 3.3. Pharmacological Modulators Effectively Regulated PKA Activity Without Toxic

**Figure 3:**
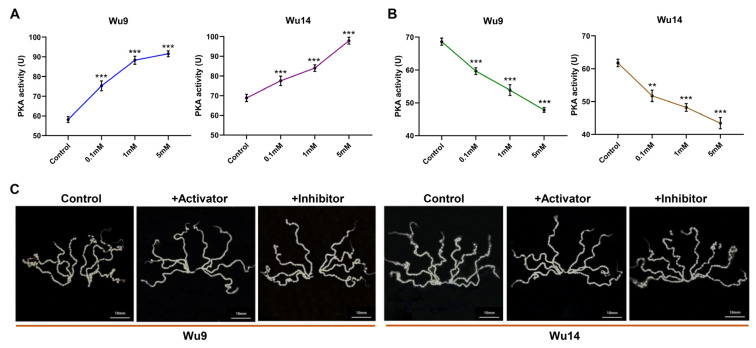
PKA activity. (**A**) Enzyme activity after activator treatment. (**B**) Enzyme activity after inhibitor treatment. (**C**) Ovarioles observation. ** *p* < 0.01; *** *p* < 0.001. The scale was 10 mm.

PKA activity assays conducted at various treatment concentrations revealed a dose-dependent response: PKA activity showed significant elevation with increasing activator concentrations, while demonstrating marked reduction with higher inhibitor doses (Figure 3A,B). Simultaneous morphological examinations of the ovarioles and egg development at different concentration gradients showed that both treatments maintained normal anatomical organization—the eight ovarioles retained their characteristic bilateral abdominal arrangement. Importantly, no significant alterations in egg developmental status or morphology were observed compared with control (Figure 3C). These findings collectively demonstrate that the chemical modulators precisely control PKA activity while maintaining physiological compatibility with normal egg development processes.

### 3.4. PKA Activity Affected Parthenogenesis Induction

**Figure 4:**
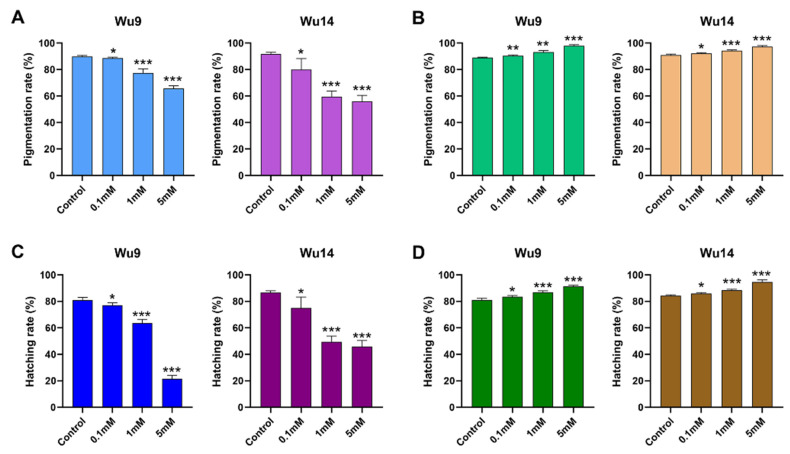
Parthenogenesis induction rate. (**A**) Pigmentation rates after activator treatment. (**B**) Pigmentation rates after inhibitor treatment. (**C**) Hatching rates after activator treatment. (**D**) Hatching rates after inhibitor treatment. * *p* < 0.05; ** *p* < 0.01; *** *p* < 0.001.

We conducted parthenogenetic induction on unfertilized eggs and quantitatively evaluated development through pigmentation and hatching rates after modulating PKA activity. Our analyses revealed that both the parthenogenetic lines Wu9 and Wu14 showed clear activity-dependent variations in developmental outcomes (Figure 4). The pharmacological inhibition of PKA consistently enhanced both pigmentation and hatching success across both lines. In contrast, PKA activation substantially hindered developmental progression, manifesting as significantly reduced pigmentation and hatching frequencies. These findings demonstrated that PKA serves as a critical regulator of parthenogenetic initiation, with its activity level exhibiting an inverse correlation with parthenogenetic induction efficiency.

### 3.5. Abnormal Elevation of PKA Activity Impeded Embryonic Development

**Figure 5:**
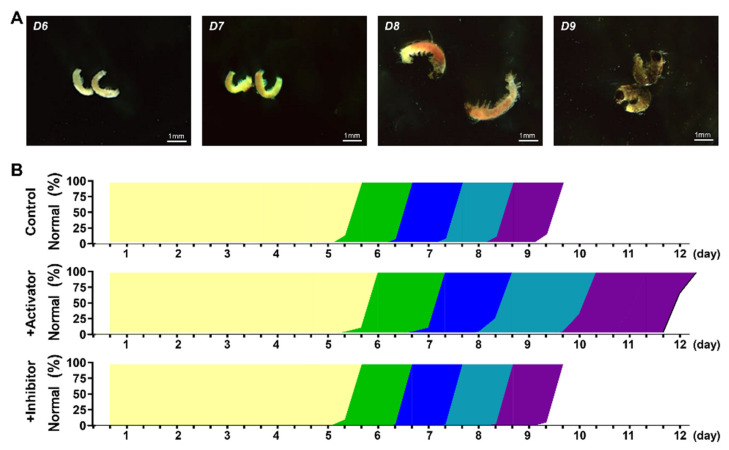
Development process of embryos. (**A**) Morphology of embryos. (**B**) Length of time. The scale was 1 mm. Yellow is D1~D5, green was D6, blue was D7, cyan was D8, purple was D9.

We performed comprehensive morphological analyses using Wu14 eggs treated with the highest concentration (5 mM) as a representative model. Our observations spanned critical developmental stages from day 6 (characterized by complete C-shaped embryo formation) to day 9 (exhibiting fully developed morphological features) (Figure 5A). Embryos in the PKA-inhibited treatment developed synchronously, whereas PKA activation caused significant developmental delays from day 6 onward compared with the control (Figure 5B). This observation suggested that, when the PKA activity exceeds a critical threshold, it led to progressive developmental arrest or even complete cessation. These results demonstrated that PKA functions as a key coordinator of multiple biological checkpoints during parthenogenesis.

### 3.6. Knockdown of PKA-C1 Gene Affected Cell Cycle Transition

**Figure 6:**
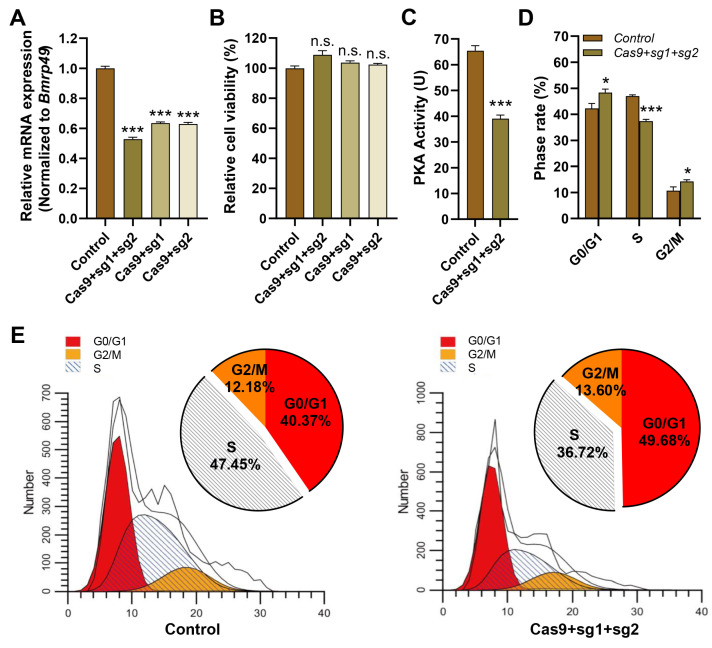
Knockdown of *PKA-C1* gene affected cell cycle. (**A**) The mRNA expression of *PKA-C1* gene. (**B**) Cell viability. (**C**) Enzyme activity. (**D**) Cell cycle. (**E**) Flow cytometry analysis. * *p* < 0.05; *** *p* < 0.001; n.s. *p* > 0.05.

We investigated the effect of PKA tetramer dysregulation on cell cycle progression, focusing on PKA-C1 as a representative catalytic subunit with core kinase functionality. We knocked down the *PKA-C1* gene to investigate its function in BmN cells. The mRNA expression of *PKA-C1* gene was significantly down-regulated after transfection, and the double-target effect was especially significant (Figure 6A). Cell viability was not affected by *PKA-C1* knockdown (Figure 6B), but PKA activity was significantly reduced (Figure 6C) and normal cell cycle progression was disrupted (Figure 6D). Tenetic perturbation induced cell cycle dysregulation in BmN cells (Figure 6E), characterized by (1) G0/G1 accumulation, (2) S phase depletion, and (3) G2/M augmentation. Although the G1-S transition showed particularly pronounced effects, the overall phenotype represents complex cell cycle dysregulation rather than simple arrest. These findings demonstrated that maintaining stable PKA activity was crucial for precise cell cycle regulation.

## 4. Discussion

Through comprehensive molecular characterization, we identified and functionally validated the PKA heterotetrameric complex in *Bombyx mori*. The R subunits function as high-affinity cAMP receptors, while the C subunits contain the conserved kinase domain responsible for phosphotransferase activity [42,43]. PKA activation is initiated through cAMP binding to R subunits, which reduces the affinity between regulatory and catalytic domains, leading to rapid release of catalytic subunits that subsequently phosphorylate nearby substrate proteins [44]. In eukaryotic cells, nearly all cAMP-mediated signaling occurs through PKA activation. The catalytic subunits transfer the γ-phosphate from ATP to serine/threonine residues on target proteins, enabling the precise regulation of protein activity through phosphorylation–dephosphorylation cycles [45].

The acquisition of oocyte developmental competence—defined as the ability to complete maturation and sustain embryonic development—constitutes an essential prerequisite for successful parthenogenetic activation [46]. Central to this process is the sophisticated interplay between the PKA signaling cascade and the core cell cycle regulators (e.g., MPF, Cdk2), which collectively orchestrate the precise spatiotemporal control of oocyte maturation events [27,28,47,48]. Our systematic functional analyses revealed that PKA serves as a critical rheostat for parthenogenesis, exhibiting dose-dependent regulatory effects. The pharmacological inhibition of PKA markedly enhanced parthenogenetic success, whereas PKA activation substantially reduced parthenogenesis rates and caused developmental delays or arrest. This aligns with established roles of PKA in regulating oocyte maturation and fertilization processes in both *Drosophila* and mammals [18,49]. Additional studies in *Xenopus* demonstrated that *PKAc* overexpression disrupts gastrulation through *RhoA* hyperactivation [50,51], collectively indicating that PKA activity must be maintained within a precise range to ensure accurate signaling during reproductive development.

The CRISPR/Cas9-mediated knockdown of *PKA-C1* in BmN cells caused severe cell cycle dysregulation, characterized by significant S phase depletion, G0/G1 and G2/M phase accumulation. These findings established that intact PKA holoenzyme function is indispensable for maintaining proper cell cycle progression in lepidopteran cells. Comparative studies in *Xenopus* revealed PKA’s negative regulation of G2/M transition [19], while yeast studies showed that PKA promotes G1/S progression through *Cln1/Cln2* phosphorylation and *Cdc28-Cyclin* complex formation [52]. In mammals, PKA facilitates G1/S transition via cAMP-dependent RhoA activation and *p27kip1* degradation [53], further establishing PKA’s conserved role in cell cycle regulation and embryonic development.

This study systematically deciphered the pivotal regulatory role of PKA in *Bombyx mori* parthenogenesis, establishing a novel “PKA–cell cycle development” axis that orchestrated the transition from oocyte maturation to parthenogenesis development. We demonstrated for the first time in insects that PKA serves as a bimodal switch governing parthenogenetic efficiency through (1) the precise modulation of cell cycle checkpoints (evidenced by cell cycle disorder upon PKA-C1 knockdown), and (2) developmental timing control (embryogenesis delay upon PKA hyperactivation). These findings redefine current models by revealing PKA’s dual-context functionality conserved in the mechanism (cAMP dependence) yet specialized in outcome (thermal induction response). Beyond fundamental insights, this work pioneered kinase-targeted reproductive engineering strategies, offering tangible applications in (1) sericulture improvement via PKA activity optimization, (2) CRISPR/Cas9-based breeding of parthenogenetic strains, (3) insect biotechnology platform development. By bridging molecular genetics with applied entomology, these discoveries open new frontiers in non-canonical reproduction research and agricultural insect management.

## 5. Conclusions

In summary, the present study elucidated the pivotal role of protein kinase A (PKA), a conserved cAMP-dependent kinase, in regulating parthenogenesis induction in *Bombyx mori*. As a heterotetrameric complex, PKA is involved in diverse cellular processes, including growth, division, and differentiation, but its role in insect parthenogenesis remains largely unknown. Here, we demonstrated that PKA activity negatively correlated with parthenogenetic efficiency, where the excessive activation of PKA delays embryonic development and its inhibition disrupts cell cycle progression. These findings highlighted the necessity of tightly regulated PKA activity for normal division and successful parthenogenesis. Given PKA’s evolutionary conservation, our results suggested its broader role as a key regulatory node in reproductive strategies across species. This work not only advances our understanding of parthenogenetic mechanisms in insects but also opens new avenues for genetic manipulation and breeding applications, particularly in enhancing asexual reproduction in economically important species.

## Figures and Tables

**Table 1:** Specific primers.

Primer Name	Primer Sequence (5′-3′)
qRT-PCR	
BmPKA-C1-F	TTCGCTGATCAACCCATTCA
BmPKA-C1-R	TGCAGCGAGGTATGAATGGA
Bmrp49-F	TCAATCGGATCGCTATGACA
Bmrp49-R	ATGACGGGTCTTCTTGTTGG
mRNA synthesis	
sgRNA-C1-F1	TAATACGACTCACTATAGGACAACTCTAACTTGTACAGTTTTAGAGCTAGAAATAGCAA
sgRNA-C1-F2	TAATACGACTCACTATAGGTTGATCAGCGAAAAAGGGGTTTTAGAGCTAGAAATAGCAA
sgRNA-R	AGCACCGACTCGGTGCCACTTTTTCAAGTTGATAACGGACTAGCCTTATTTTAACTTGCTATTTCTAGCT

**Table 2:** The protein sequence number.

Species Name	GenBank Accession Number				Order
	PKA-C1	PKA-C3	PKA-R1	PKA-R2	
*Bombyx mori*	NP_001093303.1	XP_004929251.2	NP_001093295.1	NP_001104823.1	Lepidoptera
*Bombyx mandarina*	XP_028034091.1	XP_028043672.1	XP_028029886.1	XP_028029886.1	Lepidoptera
*Drosophila melanogaster*	NP_476977.1	NP_524097.2	NP_001014593.1	NP_523671.1	Diptera
*Xenopus laevis*	NP_001080696.1	XP_018101650.1	XP_018091896.1	NP_001084637.1	Anura
*Homo sapiens*	NP_002722.1	NP_005035.1	NP_001158230.1	NP_001308911.1	Primates
*Sus scrofa*	XP_003123401.1	XP_020935351.1	XP_020941591.1	NP_999423.2	Artiodactyla
*Mus musculus*	NP_032880.1	NP_058675.1	NP_001300902.1	NP_032950.1	Rodentia
*Parasteatoda tepidariorum*	XP_015929533.1	XP_042904975.1	XP_015922609.1	XP_015906869.1	Araneae

## Data Availability

The data that support the findings of this study are available on request from the corresponding authors.
